# Supplementary material for: Antioxidant and Cytotoxic Activities of Usnea barbata (L.) F.H. Wigg. Dry Extracts in Different Solvents
Source: Plants (Basel). 2021 May 1;10(5):909. doi: 10.3390/plants10050909 (PMC8147331; doi:10.3390/plants10050909)
Supplement: Supplementary file 1 [file plants-10-00909-s001.zip › plants-1193647-supplementary.pdf]

Manuscript ID: plants-1193647

Article

Antioxidant and Cytotoxic Activities of *Usnea barbata* (L.) F.H. Wigg. Dry Extracts in Different Solvents

Violeta Popovici [1], Laura Bucur [2,*], Antoanela Popescu [2], Verginica Schroder [3], Teodor Costache [4], Dan Rambu [4], Iulia Elena Cucolea [4], Cerasela Elena Gird [5], Aureliana Caraiane [6], Daniela Gherghel [7], Gabriela Vochita [7], Victoria Badea [1]

Additional data on UHPLC-PDA method validation

An index of 240-700 nm for each analyte peak and standard solution (50 µg/ml) for ensuring peak purity was performed. Values between 1.08-1.36 were obtained, which provides a good clue that the peak of interest is pure. All the obtained data are presented in Table 1 and Figures 1-5.

Table 1. Peak-index values for UBDE in acetone, ethyl-acetate, ethanol, methanol and reference solution (usnic acid in DMSO 50 µg/mL).

Solution	Peak index
UBDE in acetone	1.28
UBDE in ethyl-acetate	1.25
UBDE in ethanol	1.31
UBDE in methanol	1.36
Standard solution (Scal) 50 µg/mL	1.08

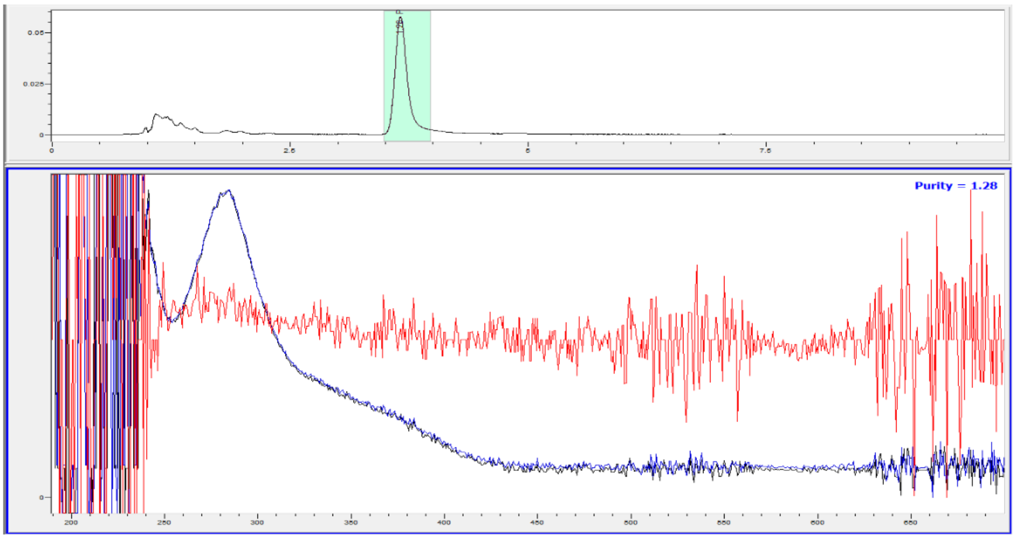

Figure 1. Peak purity of UBDE in acetone

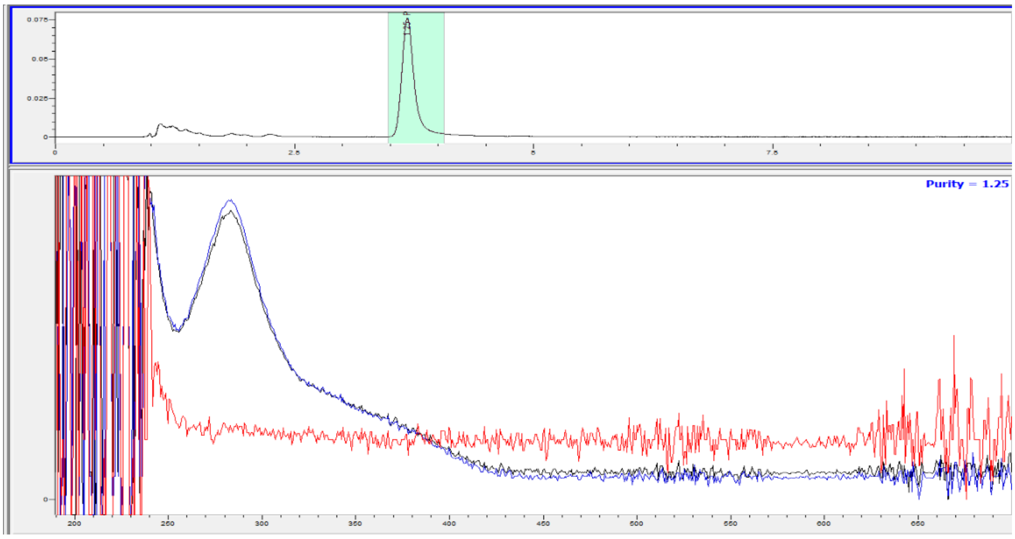

Figure 2. Peak purity of UBDE in ethyl-acetate

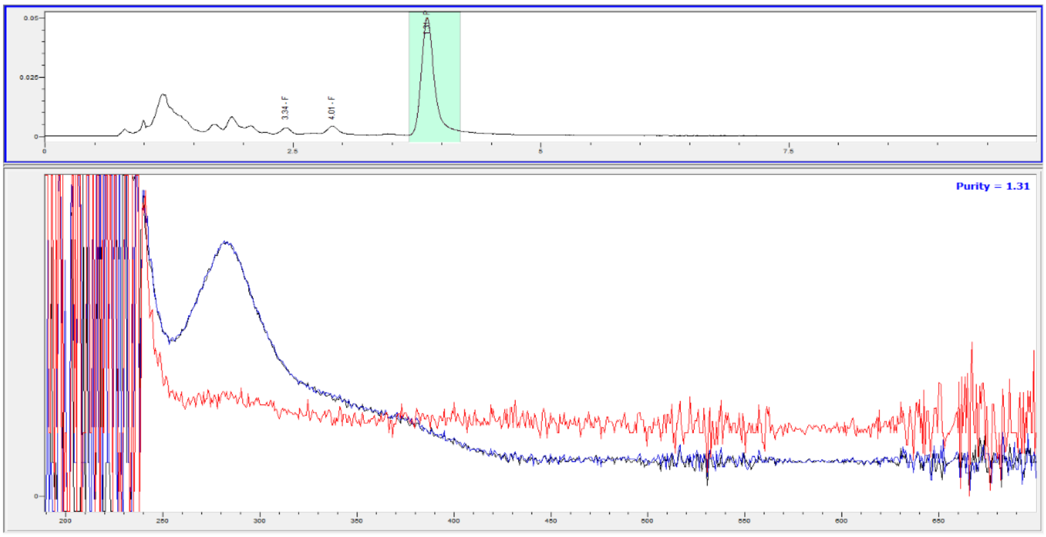

Figure 3. Peak purity of UBDE in ethanol

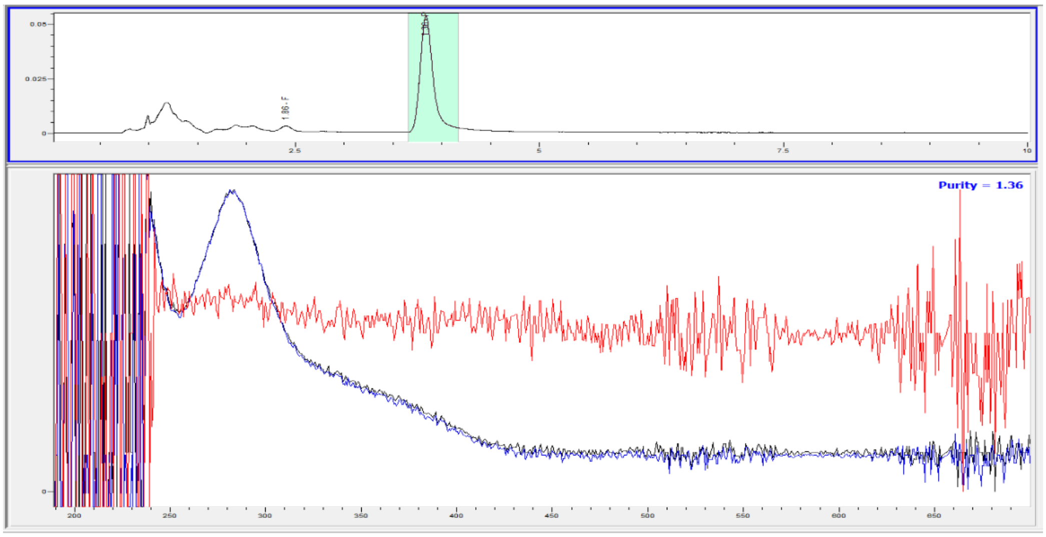

Figure 4. Peak purity of UBDE in methanol

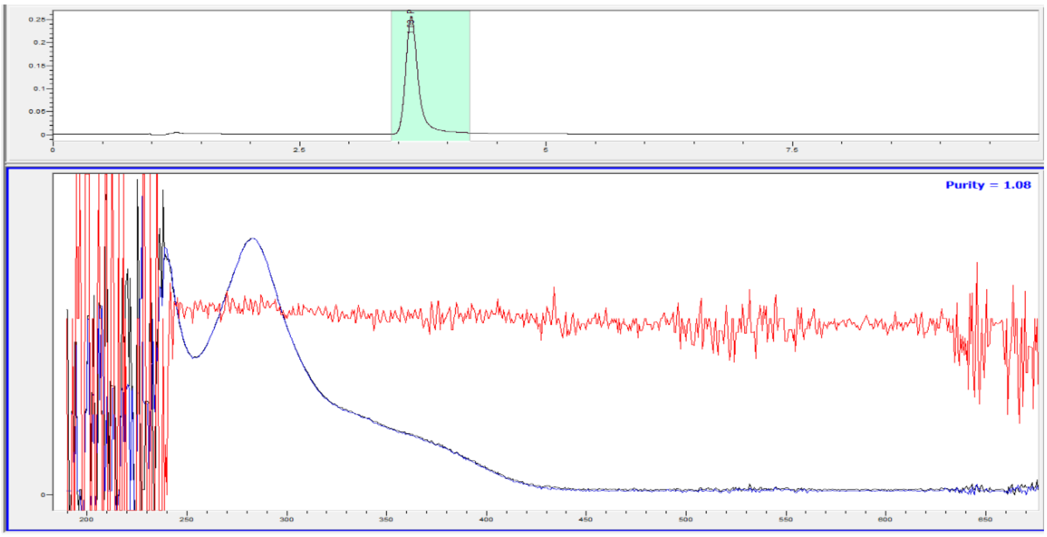

Figure 5. Peak purity of usnic acid standard solution 50 μg/mL

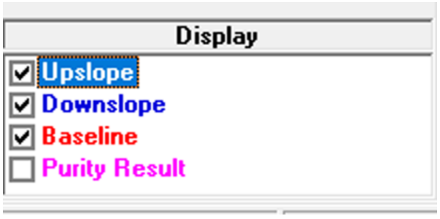

Figure 6. Display for Figures 1-5

A contour map was obtained for each one to clarify the high level of noise on 190-240 nm interval and the minor variations of indexes between references and working solutions.

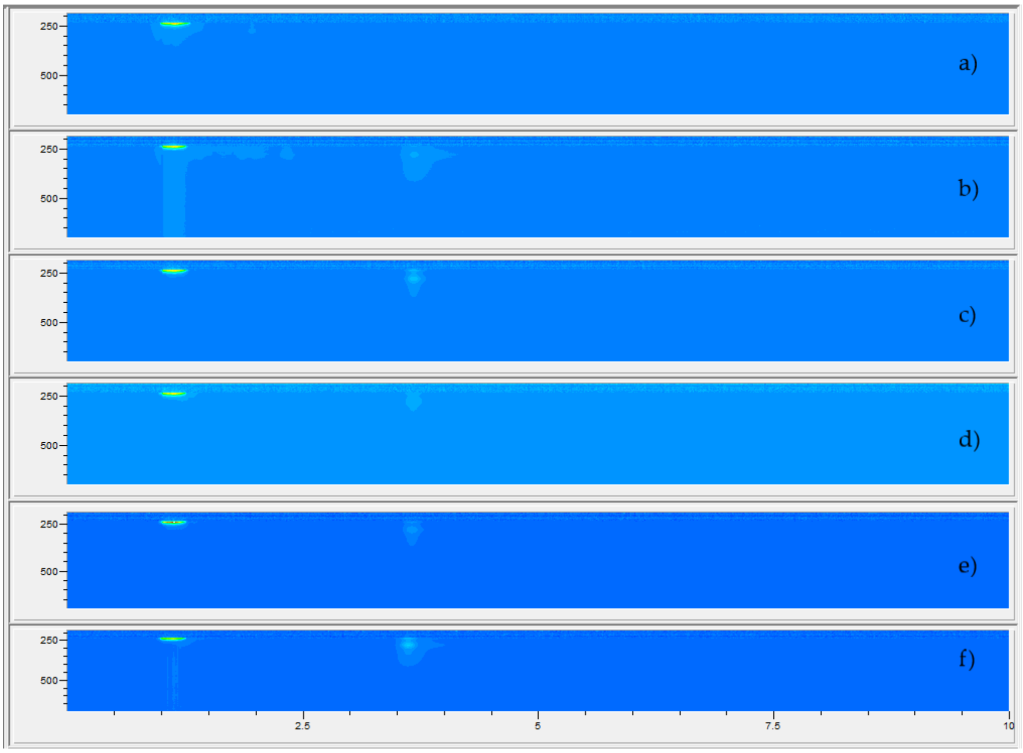

Figure 7. a) UBDE in water, b) UBDE in methanol, c) UBDE in ethyl-acetate, d) UBDE in ethanol, e) UBDE in acetone, f) usnic acid standard solution 50 μg/mL

Strong absorption bands are shown at minute 1-1.5 and 3.5-4 between 200-300nm with an intensity decay to 350, the second one corresponding to the interval in which Usnic acid shows absorption. All extracts except water extract show absorption at the given interval. On interval 190-240 is observed a noisy region on all chromatogram length corresponding to mobile phase or/and other eluting non-retained compounds. No other band are shown at different wavelengths, and contour map obtained for standard solution match the one obtained for samples in which analyte is present.

The Quality Control (QC) solutions were prepared by adding 20 µg standard-stock solution in a volumetric flask of 10 mL and completing with DMSO up to the mark.

Two samples of QC solutions (40 µg/mL) were injected at the beginning and end of the sequence to ensure analysis accuracy. Accuracy between 97.7-98.8% indicates that the analysis is highly accurate (Table 2).

Table 2. Determination of the accuracy of the method

QC Solution	Purity (%)	Theoretical concentration	Accuracy (%)
38.2874	**98**	**39.2**	97.7
38.7167			98.8
